# The ability of continuous-wave Doppler ultrasound to detect fetal growth restriction

**DOI:** 10.1371/journal.pone.0255960

**Published:** 2021-08-09

**Authors:** Ute Feucht, Helen Mulol, Valerie Vannevel, Robert Pattinson

**Affiliations:** 1 Research Centre for Maternal, Fetal, Newborn and Child Health Care Strategies, University of Pretoria, Pretoria, South Africa; 2 Maternal and Infant Health Care Strategies Research Unit, South African Medical Research Council, Pretoria, South Africa; 3 Department of Paediatrics, University of Pretoria, Pretoria, South Africa; 4 Department of Obstetrics and Gynaecology, University of Pretoria, Pretoria, South Africa; Stellenbosch University, SOUTH AFRICA

## Abstract

**Background:**

Fetal growth restriction (FGR), defined as a fetus failing to reach its genetic growth potential, remains poorly diagnosed antenatally. This study aimed to assess the ability of continuous-wave Doppler ultrasound of the umbilical artery (CWD-UmA) to detect FGR in healthy women with low-risk pregnancies.

**Methods and findings:**

This prospective longitudinal descriptive cohort study enrolled infants born to low-risk mothers who were screened with CWD-UmA between 28–34 weeks’ gestation; the resistance index (RI) was classified as normal or abnormal. Infants were assessed at 6, 10, 14 weeks, and 6 months postnatally for anthropometric indicators and body composition using the deuterium dilution method to assess fat-free mass (FFM). Neonates in the abnormal RI group were compared with those in the normal RI group, and neonates classified as small-for-gestational age (SGA) were compared with appropriate-for-gestational age (AGA) neonates.

Eighty-one term infants were included. Only 6 of 26 infants (23.1%) with an abnormal RI value would have been classified as SGA. The abnormal RI group had significantly reduced mean FFM and FFM-for-age Z-scores at 6, 10, 14 weeks, and 6 months compared with the normal RI group (P<0.015). The SGA group’s FFM did not show this consistent trend when compared to AGA FFM, being significantly different only at 6 months (P = 0.039).

The main limitation of the study was the small sample size of the infant follow-up.

**Conclusions:**

Abnormal RI obtained from CWD-UmA is able to detect FGR and is considered a useful addition to classifying the neonate only by SGA or AGA at birth.

## Introduction

Fetal growth restriction (FGR) is a major contributor to stillbirth, stunting, poor neurodevelopment, and childhood obesity as well as obesity, type-two diabetes mellitus, hypertension, and strokes in adults [1–4]. The first 1000 days, stretching from conception of the infant until two years postpartum, are generally recognised to be a critical period for laying the foundations of optimal health and development of the infant. This time period should be used to identify and treat potential risks, but FGR, defined as failure to reach full genetic growth potential, is currently not accurately diagnosed *in utero* with existing techniques, such as palpation, symphysis fundal height measurements, and imaging sonars [5]. Postnatal measurement of the fat-free mass (FFM) (i.e. lean tissue, including bone, muscle mass, and water component) determines the nutrition-related phenotypic changes brought about by FGR [6].

Doppler ultrasound of the fetal umbilical artery (UmA) can detect poor placental blood flow (measured by the resistance index—RI) and placental insufficiency, which is the major cause of FGR and stillbirths, with potential persistence of suboptimal growth and development beyond the neonatal period [4, 7–9]. The RI is calculated from the fetal UmA Doppler waveforms using the formula (systolic velocity–diastolic velocity) / systolic velocity. An increased RI is a direct measure of poor placental function and is not dependent on measuring fetal size. A systematic review of Doppler ultrasound screening of the UmA showed that its use could reduce perinatal mortality in high-risk pregnancies [10], whereas another systematic review on screening in low-risk mothers (LRM) found that it was not useful [11]. In Mamelodi in Pretoria, South Africa, screening of healthy, low-risk pregnant women using the low-cost continuous wave Doppler ultrasound (CWD) Umbiflow^TM^ device found abnormal RIs in 11.7%, including absent end diastolic flow indicating end-stage placental insufficiency in 1.5% [12]. The use of this RI information resulted in a 42% reduction in perinatal mortality when compared with similar pregnant women without such CWD-UmA assessment. In another study, children whose mothers were classified as having low-risk pregnancies and who were screened antenatally using CWD-UmA were followed up at the age of 12 years [13]. The children born to mothers who had an abnormal pulsatility index (a similar parameter to RI, but which measures the difference between the systolic and diastolic velocity divided by the mean velocity, instead of dividing by the systolic velocity) in pregnancy were found to have reduced cognitive ability and markers of future cardiovascular disease, strengthening the role that CWD-UmA might play for determining the developmental origins of health and disease.

This study examined whether a CWD-UmA cut-off determined by perinatal mortality for RI charts [8] in healthy women with pregnancies classified as low risk (the low-risk mother—LRM) can detect evidence of FGR measured by postnatal infant body composition, and compared it to the standard method of diagnosing FGR, namely assessing birth weight (BW) with respect to gestational age (GA) and classifying neonates as small-for-gestational age (SGA; conventionally as <10^th^ centile) or appropriate-for-gestational age (AGA).

## Methods

This prospective longitudinal descriptive cohort study enrolled infants with documented abnormal and normal CWD-UmA examinations during pregnancy. Study participants were recruited from the South African site of the World Health Organization (WHO) Umbiflow^TM^ International study, which commenced in October 2018 and screened pregnant mothers between 28 and 34 weeks’ gestation in five low-and middle-income countries using the low-cost CWD (Umbiflow^TM^) device. Mothers with an abnormal CWD-UmA were referred to the local tertiary hospital for conventional ultrasound including pulsed-wave Doppler and obstetric management. Upon confirmation of the abnormal Doppler values, the mother continued with regular follow-ups at the hospital, with appropriate management of the pregnancy, e.g. use of steroids, admission for delivery. If the ultrasound measured a normal RI, the mother continued antenatal care at the local clinic until delivery. The South African sites in the Tshwane District contributed 1426 study participants. Women who were enrolled in the Umbiflow^TM^ International study were approached after delivery at the two clinic sites and at the referral hospital and given a 6-week postnatal follow-up date to assess postnatal development of their infants, with inclusion of term infants only for this current analysis. Mothers were reminded telephonically of their 6-week postnatal appointment.

Inclusion criteria for the Umbiflow^TM^ International study were healthy women with low-risk singleton pregnancies (according to local antenatal care guidelines) and GA between 28 weeks 0 days and 34 weeks 0 days at the time of Umbiflow^TM^ screening who were expected to give birth in the study hospital or its catchment area. For the South African site, a woman was classified as having a low-risk pregnancy if she met the criteria for Basic Antenatal Care Plus (BANC Plus), which is the national antenatal care guideline based on the WHO recommendations for a positive pregnancy experience. Exclusion criteria included high-risk and multiple pregnancies, women who were not expected to give birth in the catchment area, and those who declined or were unable to give informed consent. Additional exclusion criteria for the infant follow-up were a mother under the age of 18 years or an infant with a severe medical condition or chromosomal or structural abnormality. Written informed consent for this study was obtained from the mothers before study inclusion of their infants.

**Fig 1:**
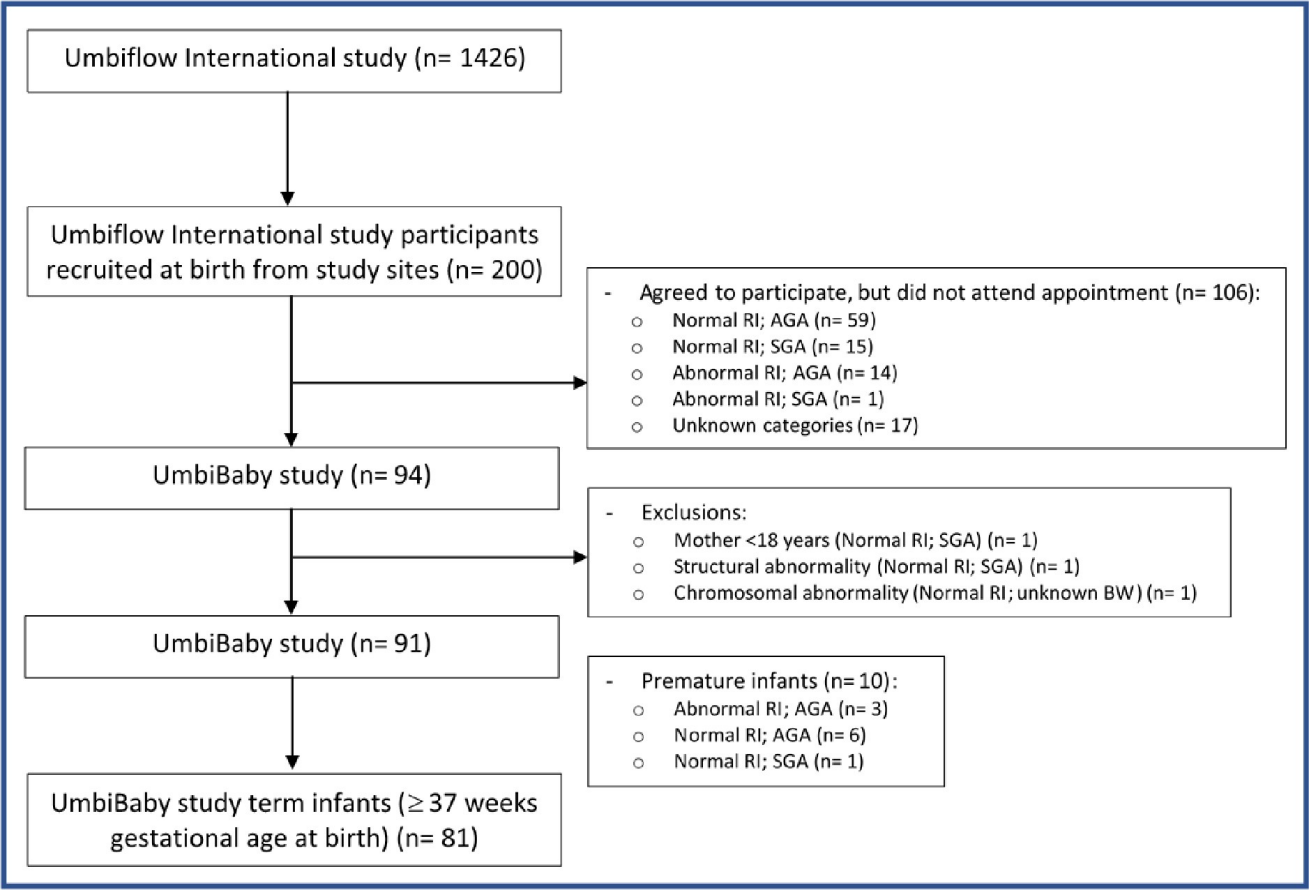
Flow diagram of infants included in the postnatal study of term infants from the Umbiflow^TM^ International Study. RI = resistance index (of the umbilical artery), AGA = appropriate-for-gestational age, SGA = small-for-gestational age, BW = birth weight.

As shown in Fig 1, a total of 200 mothers agreed to participate in the study and attend the 6-week study visit. However, only 94 mother-infant dyads came for their study visit, and of these infants, 3 were not eligible due to consent issues or medical conditions directly impacting child outcomes. Hence 91 infants were included in the postnatal follow-up, of which 10 infants were premature (<37 completed weeks gestational age at delivery), resulting in a total of 81 term infants that were included in this analysis. A sample size analysis calculated using G*Power 3.1.9.2 at an alpha level of 5% and a power of 60% revealed that a sample size of 21 per group would be sufficient for calculation of independent t-tests with a large effect size of 0.7.

Postnatal follow-up study visits were done at six weeks (w), 10w, 14w, and six months (m). The CWD-UmA result during the pregnancy was used to classify the infant into normal and abnormal RI groups, with the latter being an RI ≥75^th^ centile (including absent end diastolic flow) [8]. This cut-off of RI ≥75^th^ centile was used as this determined the best level to prevent perinatal mortality in a high-risk population. All infant assessments were blinded to the CW-UmA results in pregnancy. Other pregnancy and delivery information from the Umbiflow^TM^ International study included maternal age, maternal HIV status, GA assessment, date of delivery, infant sex, and birth anthropometry (BW, length, and head circumference (HC) as done within the routine health services) and indices (ponderal index and BW-to-HC ratio). Z-score calculation and classification of SGA and AGA at birth was done according to the INTERGROWTH-21 International Newborn Size Standards charts [14], with SGA defined as <10^th^ centile of BW-for-GA.

The main study outcomes were the infants’ nutrition-related phenotype (growth and body composition) in the first six months of life using serial anthropometry, converted to age- and sex-normalized Z-scores as per WHO growth standards [15]. No correction for GA was done during Z-score calculations as only term infants were included in this analysis. Body composition was measured by the non-invasive deuterium dilution method, which measures the infant’s total body water and from this the fat-free mass (FFM) is determined. Deuterium is a stable isotope of hydrogen and its use in nutritional studies, such as measurement of infant body composition, is considered safe at the low levels of deuterium oxide used [16]. The deuterium dilution method has been described in detail elsewhere [17], but in brief involves consumption of an accurately measured small dose (3g) of deuterium oxide, which is then allowed to equilibrate in the infant’s body. Pre- and post-dose infant saliva samples (at 2.5 hours) are collected and deuterium enrichment in the post-dose sample is compared to the pre-dose sample using a Fourier Transform Infrared Spectrometer. Body composition outcomes are absolute FFM and fat mass, % fat-free mass (%FFM) and % fat mass, fat-free mass index, fat mass index, and age- and sex-normalized FFM Z-scores (FFMAZ). FFMAZ were calculated using the LMS tables of body composition reference charts that were constructed using the same deuterium dilution method [18]. The L and S values, which represent the skewness of the values and the coefficient of variation of the values around the mean, respectively, are constant for each sex over the first six months of life, but the M value, which represents the mean, varies and did not coincide with our study’s visit time points. The published Wells M values were therefore plotted for each sex at their time points, which gave a straight line, and the equations for these straight lines were then used to obtain the exact M values for each infant in our study according to their sex and exact age at our study visit time point. Finally these L, S and calculated M values were converted to FFMAZ using Z = (((FFMkg/M)^L^)-1)/(L*S) [19].

Birth characteristics and outcome variables at each time point were assessed for normality with the Shapiro-Wilk test. Continuous variables with a normal distribution were presented as mean ± standard deviation. Continuous variables were compared between the abnormal and normal RI groups and between SGA and AGA groups using the independent t-test with Welch’s approximation, variables with a non-normal distribution were compared using the Mann-Whitney U test. Categorical variables were compared with the Chi-squared test. All statistical analyses were carried out using STATA version 13.1 (StataCorp., Texas, USA) and a P value <0.05 was considered to be statistically significant.

Permissions to conduct the study were obtained through the University of Pretoria Research Ethics Committee (reference no. 283/2019) as well as institutional permissions from the relevant health services.

## Results

The overall prevalence of an abnormal RI in the South African site of the Umbiflow^TM^ International study was 6.9% and 5.3% for the infants born at term. The baseline characteristics of the 81 term infants included in this longitudinal follow-up study are shown in Table 1, according to the normal/abnormal RI and SGA/AGA groups, respectively. There were no significant sex differences within the groups, but infants with an abnormal RI had a significantly different GA at birth, BW, weight-for-age Z-score (WAZ), HC, and weight-to-HC ratio (W/HC), and infants categorized as SGA had significant differences in BW (used for categorization), WAZ, length, length-for-age Z-score (LAZ), HC, HC-for-age Z-score (HCAZ), ponderal index, and W/HC compared to AGA infants. Only six out of the 26 infants (23.1%) with an abnormal RI value would have been classified as SGA. S1–S3 Tables compare baseline characteristics of the infant follow-up study to those of the Umbiflow^TM^ International study. The main overall difference was the percentage of abnormal RI infants, which was significantly higher in the infant follow-up study. There was no significant difference in the percentage of SGA/AGA infants between the original cohort and the infant follow-up study. Although the infant follow-up study overall was significantly different in terms of the maternal age, gestational age at birth, and many of the birth anthropometric measures compared to the original cohort, once the infants were classified into the normal/abnormal RI and SGA/AGA groups and then compared there were few significant differences between the studies, viz. the maternal age in the normal RI group and the AGA group and the percentage of abnormal RI infants in the SGA and AGA classifications.

**Table 1:** Pregnancy and birth characteristics of the study participants, grouped by resistance index of the umbilical artery and by birth weight-for-gestational age categories.

	Abnormal RI	Normal RI		SGA	AGA	
	(n = 26)	(n = 55)	P-value	(n = 14)	(n = 67)	P-value
**Maternal age** * **, y**	27.7 ± 5.2	29.5 ± 5.9	0.172	27.1 ± 5.8	29.3 ± 5.7	0.228
**Gravidity** **	2 (1–4)	2 (1–5)	0.701	2 (1–5)	2 (1–5)	0.377
**Maternal HIV status positive, n (%)**	5 (19.2%)	20 (36.4%)	0.119	6 (42.9%)	19 (28.4%)	0.285
**Infant sex, M/F**	11/15	30/25	0.304	8/6	33/34	0.591
**Gestational age at birth** * **, w**	38.3 ± 1.0	39.1 ± 1.2	**0**.**002**	38.9 ± 1.2	38.9 ± 1.2	0.981
**Birth weight (BW)** * **, g**	2818 ± 361	3171 ± 495	**0**.**002**^†^	2511 ± 250	3172 ± 441	**<0**.**001**^†^
**Birth length** * **, cm**	49.2 ± 2.2	50.4 ± 2.7	0.051	48.2 ± 1.9	50.4 ± 2.5	**0**.**001**
**Head circumference at birth** * **, cm**	33.9 ± 1.3	34.7 ± 1.6	**0**.**016**	33.7 ± 1.4	34.6 ± 1.6	**0**.**050**
**Weight-for-age Z-score** *	-0.63 ± 0.77	0.11 ± 1.09	**0**.**042**^†^	-1.67 ± 0.22	0.02 ± 0.87	**<0**.**001**^†^
**Length-for-age Z-score** *	0.47 ± 1.22	0.76 ± 1.51	0.369	-0.38 ± 1.21	0.89 ± 1.36	**0**.**002**
**Head circumference-for-age Z-score** *	0.45 ± 1.06	0.82 ± 1.27	0.177	0.07 ± 1.13	0.84 ± 1.19	**0**.**035**
**Ponderal index** ^**§**^ ^*^ **, g/cm** ^**3**^ **x100**	2.37 ± 0.32	2.49 ± 0.41	0.335^†^	2.26 ± 0.38	2.50 ± 0.38	**0**.**045**^†^
**W/HC ratio** ^**&**^ ^*^ **, g/cm**	83.2 ± 10.0	90.7 ± 11.4	**0.004**	74.5 ± 7.1	91.2 ± 10.0	**<0**.**001**
**BW for gestational age <10** ^**th**^ **centile (SGA) for RI groups, n (%)**	6 (23.1%)	8 (14.5%)	0.343	..	..	..
**Abnormal RI per SGA/AGA groups, n (%)**	..	..	..	6 (42.9%)	20 (29.9%)	0.343

*Mean ± SD

** Median (range)

^†^Mann-Whitney U test.

^§^Ponderal Index = weight (g) / length^3^ (cm) x100.

^&^W/HC ratio = Birth weight to head circumference (HC) ratio = weight (g) / HC (cm) [21].

**Abbreviations:** RI = Resistance index (of umbilical artery); SGA = small-for-gestational age; AGA = appropriate-for-gestational age; y = years; SD = standard deviation; M = male; F = female; w = weeks; g = grams; cm = centimetres; W/HC ratio = weight-to-head circumference ratio.

**Fig 2:**
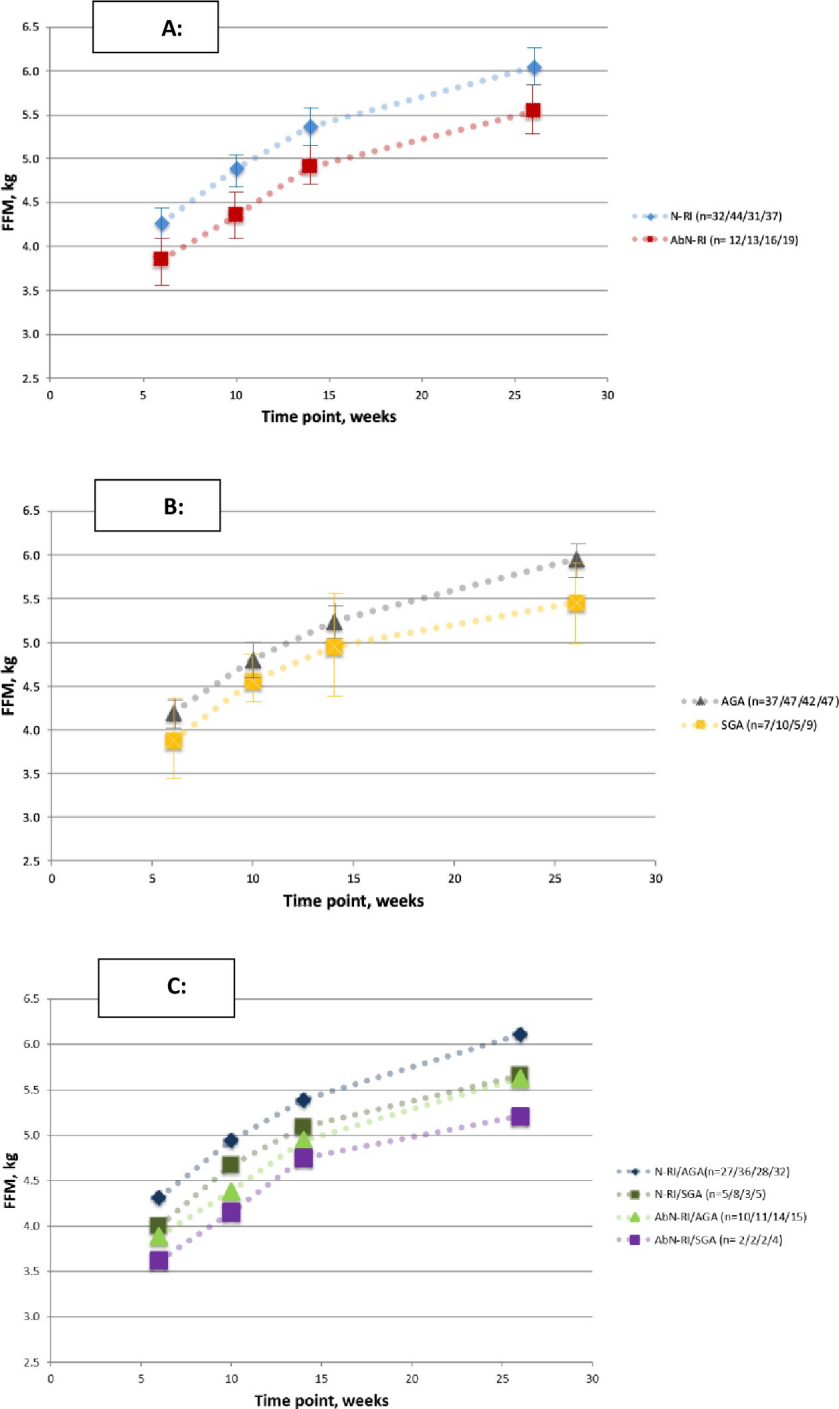
Fat-free mass in infants in the first 6 months of life. (A) Grouped according to normal and abnormal resistance indices (RI) of the umbilical artery Doppler, (B) grouped according to weight at birth in terms of appropriate-for-gestational age (AGA) and small -for-gestational age (SGA), and (C) grouped according to the umbilical artery RI and AGA/SGA classifications. Abbreviations: N-RI = normal resistance index (of umbilical artery Doppler); AbN-RI = abnormal resistance index; AGA = appropriate-for-gestational age weight at birth; SGA = small-for-gestational age weight at birth. Numbers in brackets are the numbers in the group at the 6/10/14/26 weeks’ time points, respectively. Error bars represent the 95% confidence intervals of the mean.

Tables 2 and 3 show the analysis of the infant anthropometry and body composition results at each time point, as per normal/abnormal RI and SGA/AGA groups, respectively. The absolute FFM and the FFMAZ were significantly lower in the abnormal RI group at all visits, with the FFM changes over time visually depicted in Fig 2A. Weight, length, and HC measurements were consistently smaller at all visits in the abnormal RI group, compared to the normal RI group, reaching significance for weight at 10w, 14w, and 6m, for length at 6w, 10w, and 14w, and for HC at 6w, 14w, and 6m. WAZ were significantly smaller at 10w, 14w, and 6m, LAZ at 6w, 10w, and 14w, and HCAZ at 6w and 14w.

**Table 2:** Anthropometric and body composition data at child follow-up visits, as grouped by normal and abnormal resistance index of the umbilical artery Doppler during pregnancy.

	6w visit			10w visit			14w visit			6m visit		
	Abnormal RI	Normal RI		Abnormal RI	Normal RI		Abnormal RI	Normal RI		Abnormal RI	Normal RI	
	(n = 12)	(n = 32)	P-value	(n = 13)	(n = 44)	P-value	(n = 16)	(n = 31)	P-value	(n = 19)	(n = 37)	P-value
**Age at visit** * **, w**	6.4 ± 0.5	6.3 ± 0.5	0.529^†^	10.5 ± 0.5	10.6 ± 0.5	0.818^†^	14.7 ± 1.0	14.5 ± 0.5	0.856^†^	26.2 ± 0.5	26.3 ± 1.0	0.599^†^
**Sex, M/F**	4/8	16/16	0.323	4/9	27/17	0.052	6/10	21/10	**0.047**	8/11	20/17	0.397
**SGA/AGA**	2/10	5/27	0.933	2/11	8/36	0.816	2/14	3/28	0.766	4/15	5/32	0.467
**Weight** * **, kg**	4.64 ± 0.60	4.98 ± 0.61	0.110	5.33 ± 0.65	5.97 ± 0.67	**0.005**	6.11 ± 0.79	6.80 ± 0.75	**0.007**	7.46 ± 1.00	8.28 ± 1.04	**0.007** ^†^
**Length** * **, cm**	53.1 ± 2.7	55.4 ± 2.1	**0.018**	56.8 ± 1.7	58.8 ± 2.5	**0.002**	59.7 ± 1.9	61.7 ± 2.5	**0.004**	66.1 ± 2.9	67.4 ± 3.0	0.119
**BMI** * **, kg/m** ^**2**^	16.5 ± 2.0	16.2 ± 1.6	0.706	16.5 ± 1.7	17.3 ± 1.5	0.166	17.1 ± 1.8	17.9 ± 1.7	0.165	17.1 ± 1.8	18.3 ± 2.2	0.060^†^
**HC** * **, cm**	38.2 ± 1.1	39.1 ± 1.3	**0.024**	40.1 ± 1.3	40.5 ± 0.2	0.317	41.0 ± 1.6	42.1 ± 1.0	**0.014**	43.9 ± 1.1	44.7 ± 0.9	**0.027**
**WAZ** *	-0.30 ± 1.02	0.20 ± 0.92	0.159	-0.43 ± 0.94	0.26 ± 0.86	**0.027**	-0.28 ± 0.97	0.43 ± 0.90	**0.022**	-0.21 ± 1.04	0.59 ± 1.07	**0.011**
**LAZ** *	-1.34 ± 1.29	-0.25 ± 1.01	**0.017**	-0.96 ± 0.86	-0.20 ± 1.14	**0.016**	-0.81 ± 0.89	-0.08 ± 1.25	**0.025**	-0.24 ± 1.23	0.23 ± 1.37	0.197
**WLZ** *	1.35 ± 1.51	0.68 ± 1.22	0.188	0.59 ± 1.10	0.71 ± 1.09	0.739	0.50 ± 1.09	0.74 ± 1.15	0.487	0.04 ± 1.18	0.73 ± 1.33	0.055
**BMIAZ** *	0.65 ± 1.31	0.48 ± 1.07	0.689	0.15 ± 1.07	0.53 ± 0.96	0.224	0.26 ± 1.08	0.65 ± 1.05	0.242	-0.86 ± 1.16	0.62 ± 1.34	**0.047**
**HCAZ** *	0.41 ± 0.86	1.12 ± 1.05	**0.031**	0.78 ± 1.01	0.89 ± 1.00	0.740	0.51 ± 1.06	1.24 ± 0.72	**0.026**	0.94 ± 0.83	1.40 ± 0.86	0.070
**FM** * **, kg**	0.79 ± 0.26	0.69 ± 0.26	0.269	1.03 ± 0.28	1.09 ± 0.34	0.486	1.20 ± 0.47	1.44 ± 0.45	0.098	1.88 ± 0.48	2.19 ± 0.87	0.144^†^
**FFM** * **, kg**	3.85 ± 0.39	4.26 ± 0.52	**0.008**	4.35 ± 0.46	4.89 ± 0.56	**0.007** ^†^	4.91 ± 0.43	5.36 ± 0.60	**0.013** ^†^	5.54 ± 0.63	6.05 ± 0.61	**0.007**
**FFMAZ** ^**#**^ *	-0.12 ± 0.88	0.67 ± 0.97	**0.016**	0.34 ± 0.76	1.15 ± 0.88	**0.003**	0.77 ± 0.69	1.35 ± 0.83	**0.015**	0.31 ± 0.86	1.00 ± 0.86	**0.007**
**FM** * **, %**	16.7 ± 3.6	13.8 ± 4.5	**0.035**	19.0 ± 3.9	18.1 ± 4.8	0.523	19.1 ± 5.3	21.0 ± 5.5	0.267	25.1 ± 3.8	25.9 ± 7.6	0.578
**FFM** * **, %**	83.3 ± 3.6	86.2 ± 4.5	**0.035**	81.0 ± 3.9	81.9 ± 4.8	0.523	80.9 ± 5.3	79.0 ± 5.5	0.267	74.9 ± 3.8	74.1 ± 7.6	0.578
**FMI** * **, kg/m** ^**2**^	2.8 ± 0.8	2.3 ± 0.8	0.063	3.2 ± 0.9	3.2 ± 0.9	0.898	3.3 ± 1.2	3.8 ± 1.2	0.222	4.3 ± 0. 9	4.8 ± 1.9	0.272^†^
**FFMI** * **, kg/m** ^**2**^	13.7 ± 1.6	13.9 ±1.4	0.686	13.5 ± 1.21	14.1 ± 1.2	0.108	13.8 ± 1.1	14.1 ± 1.3	0.391	12.7 ± 1.4	13.3 ± 1.4	0.073^†^

* Mean ± SD

^#^Fat-free mass-for age Z-score calculations per age and sex done according to Wells *et al*. [18]

^†^Mann-Whitney U test.

**Abbreviations:** RI = resistance index (of umbilical artery); w = weeks; m = months; M = male; F = female; SGA = small-for-gestational age; AGA = appropriate-for-gestational age; kg = kilograms; cm = centimetres; BMI = body mass index; kg/m^2^ = kilogram per meter squared; HC = head circumference; WAZ = weight-for-age Z-score; LAZ = length-for-age Z-score; WLZ = weight-for-length Z-score; BMIAZ = BMI-for-age Z-score; HCAZ = head circumference-for-age Z-score; FM = fat mass; FFM = fat-free mass; FFMAZ = fat-free mass-for-age Z-score; FM% = fat mass percentage; FFM% = fat-free mass percentage; FMI = fat mass index; FFMI = fat-free mass.

**Table 3:** Anthropometric and body composition data at child follow-up visits, as grouped by birth weight categorization into small-for-gestational age and appropriate-for-gestational age.

	6w visit			10w visit			14w visit			6m visit		
	SGA	AGA		SGA	AGA		SGA	AGA		SGA	AGA	
	(n = 7)	(n = 37)	P-value	(n = 10)	(n = 47)	P-value	(n = 5)	(n = 42)	P-value	(n = 9)	(n = 47)	P-value
**Age at visit** * **, w**	6.6 ± 0.5	6.3 ± 0.5	0.241^†^	10.7 ± 0.5	10.5 ± 0.5	0.627^†^	14.7 ± 0.5	14.6 ± 1.0	0.394^†^	26.0 ± 0.5	26.4 ± 1.0	0.258^†^
**Sex, M/F**	4/3	16/21	0.498	6/4	25/22	0.695	4/1	23/19	0.281	5/4	23/24	0.716
**Abnormal RI/ normal RI**	2/5	10/27	0.933	2/8	11/36	0.816	2/3	14/28	0.766	4/5	15/32	0.467
**Weight** * **, kg**	4.47 ± 0.53	4.97 ± 0.61	**0.049**	5.52 ± 0.54	5.89 ± 0.73	0.081	6.20 ± 0.80	6.61 ± 0.83	0.322	7.47 ± 1.08	8.10 ± 1.08	0.170^†^
**Length** * **, cm**	52.9 ± 2.7	55.1 ± 2.2	0.079	56.5 ± 1.7	58.7 ± 2.4	**0.003**	59.0 ± 3.1	61.2 ± 2.4	0.180	66.0 ± 2.3	67.1 ± 3.1	0.205
**BMI** * **, kg/m** ^**2**^	15.9 ± 0.7	16.3 ± 1.8	0.284	17.3 ± 1.8	17.0 ± 1.5	0.681	17.9 ± 2.5	17.6 ± 1.6	0.838	17.2 ± 2.2	18.0 ± 2.1	0.366^†^
**HC** * **, cm**	38.1 ± 1.2	39.0 ± 1.3	0.111	39.9 ± 1.0	40.5 ± 1.4	0.179	41.4 ± 1.3	41.8 ± 1.4	0.586	43.9 ± 1.1	44.5 ± 1.0	0.149
**WAZ** *	-0.77 ± 0.81	0.22 ± 0.91	**0.015**	-0.39 ± 0.75	0.21 ± 0.92	**0.044**	-0.48 ± 0.96	0.27 ± 0.96	0.154	-0.29 ± 1.30	0.44 ± 1.06	0.144
**LAZ** *	-1.66 ± 1.23	-0.34 ± 1.07	**0.029**	-1.35 ± 0.51	-0.17 ± 1.11	**<0.001**	-1.51 ± 1.44	-0.19 ± 1.08	0.106	-0.37 ± 0.84	0.16 ± 1.40	0.146
**WLZ** *	1.14 ± 0.80	0.81 ± 1.40	0.402	1.10 ± 1.31	0.59 ± 1.02	0.275	1.00 ± 1.89	0.61 ± 1.02	0.675	-0.03 ± 1.71	0.59 ± 1.22	0.319
**BMIAZ** *	0.21 ± 0.57	0.58 ± 0.20	0.216	0.53 ± 1.20	0.42 ± 0.95	0.798	0.55 ± 1.60	0.51 ± 1.01	0.960	-0.12 ± 1.74	0.48 ± 1.22	0.347
**HCAZ** *	0.13 ± 0.82	1.08 ± 1.02	**0.020**	0.43 ± 0.60	0.96 ± 1.04	**0.039**	0.53 ± 0.72	1.05 ± 0.94	0.185	0.86 ± 0.81	1.32 ± 0.87	0.145
**FM** * **, kg**	0.57 ± 0.25	0.75 ± 0.25	0.124	0.95 ± 0.34	1.11 ± 0.32	0.194	1.24 ± 0.40	1.37 ± 0.47	0.514	1.95 ± 0.58	2.11 ± 0.80	0.569^†^
**FFM** * **, kg**	3.89 ± 0.50	4.20 ± 0.51	0.173	4.57 ± 0.36	4.81 ± 0.62	0.266^†^	4.95 ± 0.49	5.24 ± 0.59	0.334^†^	5.46 ± 0.58	5.96 ± 0.65	**0.039**
**FFMAZ** ^**#**^ *	-0.21 ± 0.93	0.58 ± 0.98	0.072	0.57 ± 0.53	1.05 ± 0.96	**0.036**	0.56 ± 0.69	1.23 ± 0.82	0.088	0.13 ± 0.95	0.88 ± 0.95	**0.023**
**FM** * **, %**	12.8 ± 4.7	15.0 ± 4.3	0.278	16.9 ± 4.8	18.6 ± 4.6	0.318	19.7 ± 3.8	20.4 ± 5.6	0.735	25.9 ± 4.7	25.6 ± 6.8	0.899
**FFM** * **, %**	87.2 ± 4.7	85.0 ± 4.3	0.278	83.1 ± 4.8	81.4 ± 4.6	0.318	80.3 ± 3.8	79.6 ± 5.6	0.735	74.2 ± 4.7	74.4 ± 6.8	0.899
**FMI** * **, kg/m** ^**2**^	2.0 ± 0.8	2.5 ± 0.8	0.226	3.0 ± 1.1	3.2 ± 0.9	0.566	3.6 ± 1.2	3.6 ± 1.2	0.907	4.5 ± 1.4	4.7 ± 1.7	0.680^†^
**FFMI** * **, kg/m** ^**2**^	13.9 ± 1.0	13.8 ± 1.5	0.938	14.3 ± 1.1	13.9 ± 1.3	0.310	14.3 ± 1.7	14.0 ± 1.2	0.703	12.6 ± 1.3	13.2 ± 1.4	0.163^†^

*Mean ± SD

^#^Fat-free mass-for age Z-score calculations per age and sex done according to Wells *et al*. [18]

^†^Mann-Whitney U test.

**Abbreviations:** RI = resistance index (of umbilical artery); w = weeks; m = months; M = male; F = female; SGA = small-for-gestational age; AGA = appropriate-for-gestational age; kg = kilograms; cm = centimetres; BMI = body mass index; kg/m^2^ = kilogram per meter squared; HC = head circumference; WAZ = weight-for-age Z-score; LAZ = length-for-age Z-score; WLZ = weight-for-length Z-score; BMIAZ = BMI-for-age Z-score; HCAZ = head circumference-for-age Z-score; FM = fat mass; FFM = fat-free mass; FFMAZ = fat-free mass-for-age Z-score; FM% = fat mass percentage; FFM% = fat-free mass percentage; FMI = fat mass index; FFMI = fat-free mass index; SD = standard deviation.

Weight-for-length Z-scores (WLZ), body mass index (BMI), and BMI-for-age Z-scores (BMIAZ) were not significantly different at all time points, except for BMIAZ at 6m.

When comparing SGA and AGA infants, FFM was lower in the SGA infants throughout (Fig 2B), but significant only at 6m, while the FFMAZ was significantly less in the SGA group at 10w and 6m. Weight, length, and HC measurements were smaller at all visits in the SGA infants, but only reaching significance for weight at 6w and length at 10w, and none for HC. WAZ, LAZ, and HCAZ were significantly smaller in SGA infants at 6w and 10w, but not thereafter, and WLZ, BMI, and BMIAZ were not significantly different at all time points.

A visual comparison of the FFM over time, combining the RI and SGA/AGA groups, is depicted in Fig 2C, showing the normal RI/AGA group, normal RI/SGA group, abnormal RI/AGA, and abnormal RI/SGA group in decreasing order. Table 4 shows the ANOVA analysis of the FFM at each time point, which revealed a significant difference between the four groups at 6 and 10 weeks and at 6 months. Post-hoc analysis showed significant differences between the N-RI/AGA and AbN-RI/AGA groups at 10 weeks (P = 0.028) and between the N-RI/AGA and AbN-RI/SGA groups at 6 months (P = 0.045).

**Table 4:** ANOVA analysis with post-hoc comparisons of the infants’ fat-free mass in the first 6 months of life according to the umbilical artery RI and AGA/SGA classifications.

P-values for ANOVA and post-hoc comparisons	6 weeks	10 weeks	14 weeks	6 months
**ANOVA**	0.046*	0.013*	0.059	0.010*
**N-RI/AGA vs N-RI/SGA**	>0.999	>0.999	>0.999	0.763
**N-RI/AGA vs AbN-RI/AGA**	0.145	0.028*	0.093	0.086
**N-RI/AGA vs AbN-RI/SGA**	0.348	0.313	0.711	0.045*
**AbN-RI/AGA vs AbN-RI/SGA**	>0.999	>0.999	>0.999	>0.999
**AbN-RI/AGA vs N-RI/SGA**	>0.999	>0.999	>0.999	>0.999
**AbN-RI/SGA vs N-RI/SGA**	>0.999	>0.999	>0.999	>0.999

**Abbreviations:** N-RI = normal resistance index (of umbilical artery Doppler); AbN-RI = abnormal resistance index; AGA = appropriate-for-gestational age weight at birth; SGA = small-for-gestational age weight at birth.

**Fig 3:**
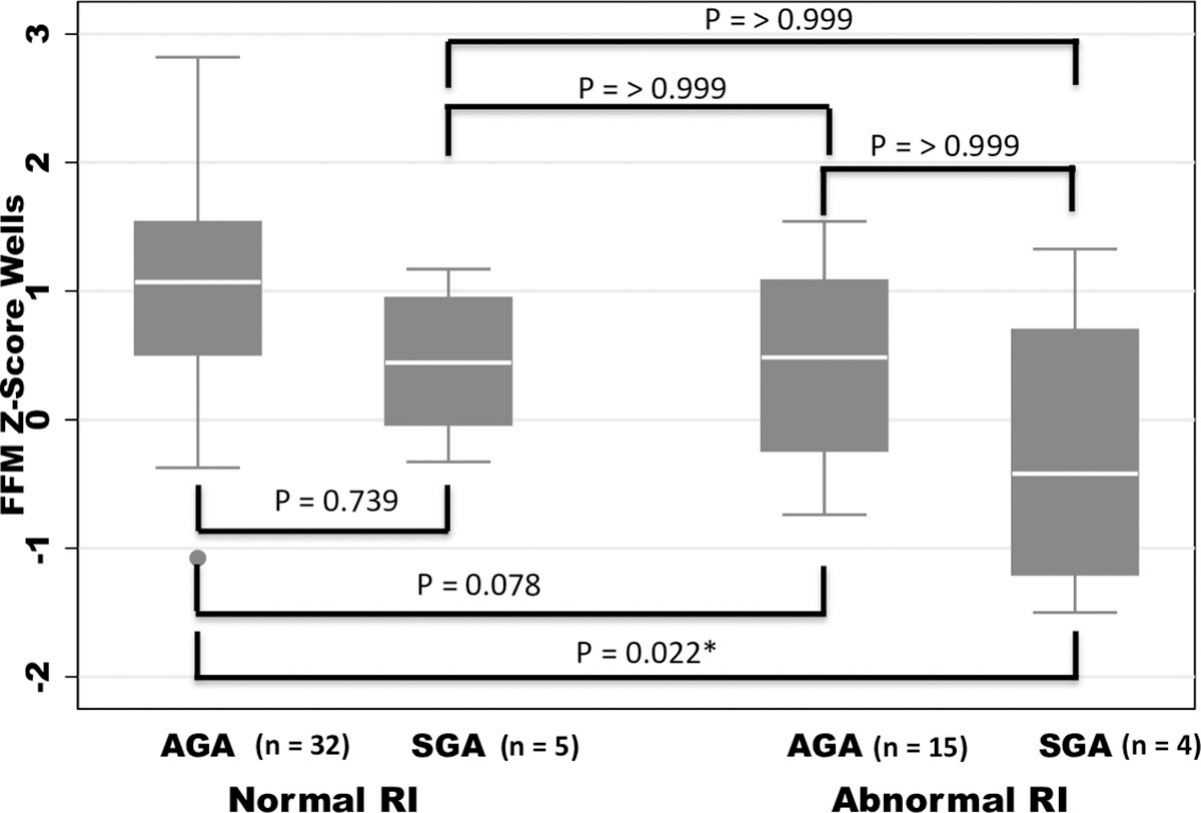
Fat-free mass Z-scores at age 6 months according to normal/abnormal RI and AGA/SGA classifications. *Denotes significant difference between groups. Abbreviations: FFM = fat-free mass; AGA = appropriate-for-gestational age weight at birth; SGA = small-for-gestational age weight at birth; Normal RI = normal resistance index (of the umbilical artery Doppler); Abnormal RI = abnormal resistance index.

Fig 3 shows a box plot of the FFMAZ at 6m for the four groups mentioned above. ANOVA analysis revealed a statistically significant difference between the 4 groups (P = 0.006). Post hoc analysis with the Bonferroni test revealed a significant difference only between the normal RI/AGA and the abnormal RI/SGA groups (P = 0.022), showing that both the AbN-RI and SGA are at risk for altered postnatal growth.

## Discussion

This study demonstrated that in low-risk pregnancies an abnormal CWD-UmA RI at 28–34 weeks’ gestation detects FGR and is therefore considered to be useful in addition to measuring neonatal size. The FFM, and the age- and sex-adjusted Z-scores thereof, were significantly lower at all the measured time points until age six months in infants with abnormal RIs, compared to those with normal RIs, indicating continued altered post-natal growth. More importantly, more than three quarters of the infants with abnormal RIs would not have been identified as growth restricted using standard growth charts and the SGA/AGA classification. The results showed that both abnormal CWD-UmA RI and SGA are associated with lower FFM at all time points and lower FFMAZ at 6 months of age compared to the normal CWD-UmA RI and AGA infants, respectively and both methods identify infants who are at risk of altered postnatal growth. Infants falling into both the abnormal RI and SGA groups had the lowest FFM and FFMAZ at 6 months, with this group being most at risk of altered postnatal growth. At all the time points there were no significant differences in FFM between the AbN-RI/AGA and AbN-RI/SGA infants and the N-RI/AGA and N-RI/SGA infants, showing that the SGA/AGA classification alone did not seem to identify possible FGR shown by a reduced FFM. Interestingly, there was also no significant difference between the FFM of the AbN-RI/AGA and N-RI/SGA infants at all time points, showing that the impact in terms of the infant’s FFM postpartum was similar in infants who had either an abnormal CWD-UmA RI in pregnancy or were SGA at birth.

Placental insufficiency leads to asymmetric FGR due to redistribution of fetal blood flow and fetal brainsparing, which can eventually lead to stillbirths, the catastrophic end result of severe placental insufficiency [1]. A lower W/HC ratio has previously been shown to indicate asymmetrical FGR, and in our study newborns in the abnormal RI group had significantly lower W/HC ratios compared to newborns in the normal RI group [20, 21]. In terms of postnatal growth, Z-scores for weight, length, and HC in SGA babies in our cohort were significantly lower than the AGA group up to 10 weeks, but not thereafter. In contrast the infants in the abnormal RI group had lower anthropometry over the six-month period, especially for weight and HC, despite the majority (>75%) of the abnormal RI group classifying as AGA at birth.

SGA babies are just small by BW, and although associated with poor outcomes, size alone does not define FGR, which is defined as failure to reach the full genetic growth potential [4, 7, 22]. The complications associated with FGR are well recognised, and the degree of poor growth probably relates to the degree of risk, with a fetus who crosses growth centiles at higher risk than one who follows the growth centiles [5]. This has been demonstrated by using customised ultrasound growth charts [23]. It appears a single RI measurement of the umbilical artery between 28–34 weeks’ gestation can do the same as the customised ultrasound growth curves. However, a single measure of neonatal size misses the majority of at-risk fetuses/infants as a neonatal weight assessment alone lacks the hindsight of the fetal growth trajectory *in utero*, as well as the future growth trajectory postnatally, and whether or not the infant has achieved its full genetic growth potential.

Umbiflow^TM^ has previously been shown to be a suitable instrument to screen a large number of low-risk pregnant women by low-level health workers, as it is an inexpensive and mobile apparatus that gives immediate results, facilitating prompt referral to next-level obstetric care. This makes it feasible for widespread use in low- and middle-income countries. It has already been established in the identification of fetuses at risk of stillbirth and reduction in perinatal mortality [12], however, the impact on the infants’ postpartum growth had not previously been studied. The study results showed that routine CWD-UmA measurements would be a useful addition to routine anthropometry at birth as it could identify additional infants with evidence of altered postpartum growth. This is of great importance as it has the potential to open up the first 1000 days to interventions to prevent suboptimal growth in at-risk infants with FGR.

The main strength of this paper is the use of a solid postnatal measurement standard for assessing FGR. This assessment of body composition, including FFM, can only be used in research settings, but the information gained is a major value-add. Another advantage is that LRMs were recruited, thereby targeting a group of pregnant women often ignored within the clinical care setting, where a simple test (Umbiflow^TM^) could reliably identify FGR. Although the risk of fetal compromise is higher in women with pregnancy complications, the absolute number of compromised fetuses is greatest from the LRM group. By measuring the neonate, the errors inherent in calculating the Estimated Fetal Weight were removed, giving the best possible groups for SGA/AGA neonates. Imaging ultrasound only gives a weight estimate and would be even less accurate than neonatal measurements. Study weaknesses include that not all women and infants were followed up, which could lead to bias, and the sample size of the infant follow-up cohort, which was limited by budgetary constraints, and resulted in a low number of SGA infants being included in the cohort, which resulted in a reduced statistical power for some of the analyses, especially for the SGA/AGA classification. Birth anthropometry measurements were done within the routine health services, possibly limiting the accuracy. The difference in mean gestational age at birth of 0.8 weeks in the AbN-RI compared to the N-RI infants may have accounted for some of the differences in anthropometry at birth and in the early postnatal period in this classification. The follow-up study was not an accurate representation of the infants in the original cohort as a significantly higher number of AbN-RI infants were enrolled in the study, which may have caused some bias and limit the generalizability of the findings. However, we consider that the purpose of the study was to compare the outcomes of infants with a N-RI and AbN-RI in a low-risk pregnant population, rather than to accurately reflect the participants in the original cohort.

Accepting a single measure of the umbilical artery RI as a red flag for FGR is only the beginning of this research. The most appropriate CWD-UmA RI cut-off for normal and abnormal; the most appropriate GA at which the screening is performed; and the need for a second screening in fetuses whose initial RIs were normal needs to be determined. The health system changes required to introduce such a large-scale screening system will be a challenge especially on the referral services as additional at-risk fetuses who were not detected before by other means, will now be referred for assessment. Furthermore, the neonatal services will be under pressure from additional neonatal admissions. The value of the abnormal RI in predicting intrapartum fetal distress will also need to be carefully assessed as it has the potential to lead to overreaction. Initially, the management will be about the correct timing of delivery of the fetus, but hopefully interventions can be developed that can improve the placental function *in utero*, therefore preventing FGR. Once delivered, the pediatric staff will need to determine what, if any, intervention is necessary to improve infant growth and how to change the metabolic profile of the infant to prevent adult disease. Finally, the ideal would be to determine the aetiology of the poor placental function and develop methods to prevent it from developing in the first place.

We are not the first to question the role of SGA 10^th^ weight centile as the definition of growth restriction [7, 21], nor to be concerned about the long-term effects of abnormal umbilical artery blood flow [13]. Clear associations between prematurity, low birth weight and SGA, and chronic non-communicable diseases in adult life have been demonstrated [24]. Detecting FGR is a high priority and Hirst et al. reported that the greatest risk of stillbirths was in babies not suspected to have been growth-restricted antenatally and that current strategies to detect FGR are not very effective [25]. Lee et al. commented that the burden of SGA births is very high in LMICs and that there should be urgent prioritization to implement effective interventions for babies born too small or too soon in order to increase survival and reduce disability, stunting, and non-communicable diseases [26]. However, strategies to detect SGA babies in LMICs using a two-step routine imaging sonar have not been found to be effective [27]. Perhaps screening pregnant women using readily scalable, low-cost CWD might be an effective method to detect fetal growth restriction in LMICs and adding the use of CWD-UmA has the potential to greatly improve the risk assessment. We therefore suggest routine screening with CWD-UmA at 28–34 weeks’ gestation to detect poor placental blood flow (measured by the RI) and identify the truly growth restricted fetus, with reduced FFM, that will not reach its genetically determined potential weight, before or after birth, and, without intervention, will have an increased risk of stillbirth. Further, such screening potentially gives us the opportunity to maximize fetal health/wellbeing antenatally, and identify infants needing special care postnatally. CWD-UmA screening therefore gives the clinician an additional tool to identify the LRM with the high-risk fetus, which is currently a major clinical challenge.

### Conclusion

Fetuses detected with an abnormal RI between 28–34 weeks’ gestation had serially significantly less FFM up to 6 months than fetuses with a normal RI, whereas the SGA neonates did not show this consistent trend when compared to AGA neonates. The implications are that it is possible to detect FGR in LRMs antenatally using CWD-UmA, and it may be a useful addition to assessing fetal or neonatal size. Therefore, its use as a screening method for FGR in place of palpation, symphysis fundal height measurements, and imaging ultrasound should be explored further.

## Supporting information

S1 Table Pregnancy and birth characteristics of the infant follow-up study compared to the Umbiflow^TM^ International participants, including grouping by resistance index of the umbilical artery and by birth weight-for-gestational age categories. (DOCX)

S2 Table Pregnancy and birth characteristics of the infant follow-up study compared to the Umbiflow^TM^ International participants, grouped by resistance index of the umbilical artery categories. (DOCX)

S3 Table Pregnancy and birth characteristics of the infant follow-up study compared to the Umbiflow^TM^ International participants, grouped by birth weight-for-gestational age categories. (DOCX)

S1 Dataset (XLSX)

## Abbreviations

AGA: appropriate-for-gestational age
BMI: body mass index
BMIAZ: BMI for age Z-score
BW: birth weight
CWD-UmA: continuous-wave Doppler ultrasound of the umbilical artery
FFM: fat-free mass
FFMAZ: fat-free mass-for-age Z-score
FGR: fetal growth restriction
GA: gestational age
HC: head circumference
HCAZ: head circumference-for-age Z-score
LAZ: length-for-age Z-score
LRM: low-risk mothers
m: month
RI: resistance index
SGA: small-for-gestational age
UmA: umbilical artery
w: week
WAZ: weight-for-age Z-score
WHO: World Health Organization
WLZ: weight-for-length Z-score
W/HC: weight to head circumference ratio
