# Supplementary material for: The ability of continuous-wave Doppler ultrasound to detect fetal growth restriction
Source: PLoS One. 2021 Aug 9;16(8):e0255960. doi: 10.1371/journal.pone.0255960 (PMC8351973; doi:10.1371/journal.pone.0255960)
Supplement: S1 Table — (DOCX) [file pone.0255960.s001.docx]

**Supplementary Table 1: Pregnancy and birth characteristics of the infant follow-up study compared to the Umbiflow^TM^ International participants, including grouping by resistance index of the umbilical artery and by birth weight-for-gestational age categories.**

|  | **Umbiflow International study** | **UmbiBaby study** | **P-value** |
| --- | --- | --- | --- |
|  | **(n=1130)** | **(n=81)** |  |
| **Maternal age^*^, y** | 27.6 ± 5.1 | 28.9 ± 5.7 | **0.049** |
| **Gravidity**** | 2 (1-12) | 2 (1-5) | 0.693^†^ |
| **Maternal HIV status positive, n (%)** | 341 (33.8%) | 25 (30.9%) | 0.577 |
| **Infant sex, M/F** | 566/560 | 41/40 | 0.943 |
| **Gestational age at birth*, w** | 39.3 ± 1.4 | 38.9 ± 1.2 | **0**.**005** |
| **Birth weight (BW)*, g** | 3175 ± 553 | 3057 ± 483 | **0**.**038** |
| **Birth length*, cm** | 50.6 ± 2.6 | 50.0 ± 2.6 | **0.048** |
| **Head circumference at birth*, cm** | 34.4 ± 1.5 | 34.4 ± 1.6 | >0.999 |
| **Weight-for-age Z-score*** | -0.14 ± 0.99 | -0.59 ± 1.05 | **<0.001** |
| **Length-for-age Z-score*** | 0.87 ± 1.44 | 0.16 ± 1.39 | **<0.001** |
| **Weight-for-length Z-score*** | -1.18 ± 1.48 | -1.21 ± 1.52 | 0.864 |
| **Head circumference-for-age Z-score*** | 0.59 ± 1.22 | 0.15 ± 1.27 | **0.003** |
| **BW-for-gestational age <10^th^ centile (SGA), n (%)** | 124 (11.0%) | 14 (17.3%) | 0.071 |
| **Abnormal RI, n (%)** | 60 (5.3%) | 26 (32.1%) | **<0.001** |
| **Abnormal RI/ AGA, n (%)** | 48 (4.4%) | 20 (24.7%) | **<0.001** |
| **Abnormal RI/ SGA, n (%)** | 12 (1.1%) | 6 (7.4%) | **<0.001** |
| **Normal RI/ AGA, n (%)** | 923 (84.3%) | 47 (58.0%) | **<0.001** |
| **Normal RI/ SGA, n (%)** | 112 (10.2%) | 8 (9.9%) | 0.923 |

* Mean ± SD; ** Median (range); ^†^ Mann-Whitney U test

**Abbreviations:** y= years; n= number; M= male; F= female; w= weeks; g= grams; cm= centimetres; BW= birth weight; RI= Resistance index (of umbilical artery); SGA= small-for-gestational age; AGA= appropriate-for-gestational age; SD= standard deviation
