# Supplementary material for: The ability of continuous-wave Doppler ultrasound to detect fetal growth restriction
Source: PLoS One. 2021 Aug 9;16(8):e0255960. doi: 10.1371/journal.pone.0255960 (PMC8351973; doi:10.1371/journal.pone.0255960)
Supplement: S2 Table — (DOCX) [file pone.0255960.s002.docx]

**Supplementary Table 2: Pregnancy and birth characteristics of the infant follow-up study compared to the Umbiflow^TM^ International participants, grouped by resistance index of the umbilical artery categories.**

|  | **Abnormal RI** | |  | **Normal RI** | |  |
| --- | --- | --- | --- | --- | --- | --- |
|  | **Umbiflow International** | **UmbiBaby** | **P-value** | **Umbiflow International** | **UmbiBaby** | **P-value** |
|  | **(n=60)** | **(n=26)** |  | **(n=1070)** | **(n=55)** |  |
| **Maternal age^*^, y** | 27.6 ± 5.2 | 27.7 ± 5.2 | 0.935 | 27.6 ± 5.1 | 29.5 ± 5.9 | **0.023** |
| **Gravidity**** | 3 (1-12) | 2 (1-4) | 0.437^†^ | 2 (1-7) | 2 (1-5) | 0.669^†^ |
| **Maternal HIV status positive, n (%)** | 17 (28.3%) | 5 (19.2%) | 0.305 | 324 (30.2%) | 20 (36.4%) | 0.319 |
| **Infant sex , M/F** | 26/34 | 11/15 | 0.916 | 540/526 | 30/25 | 0.564 |
| **Gestational age at birth*, w** | 38.6 ± 1.2 | 38.3 ± 1.0 | 0.235 | 39.4 ± 1.4 | 39.1 ± 1.2 | 0.078 |
| **Birth weight* (BW), g** | 2917 ± 422 | 2818 ± 361 | 0.273 | 3190 ± 556 | 3171 ± 495 | 0.784 |
| **Length*, cm** | 49.6 ± 2.5 | 49.2 ± 2.2 | 0.461 | 50.6 ± 2.6 | 50.4 ± 2.7 | 0.594 |
| **Head circumference*, cm** | 34.0 ± 1.8 | 33.9 ± 1.3 | 0.773 | 34.5 ± 1.5 | 34.7 ± 1.6 | 0.368 |
| **Weight-for-age Z-score*** | -0.48 ± 0.89 | -0.63 ± 0.77 | 0.433 | -0.12 ± 1.00 | 0.11 ± 1.09 | 0.131 |
| **Length-for-age Z-score*** | 0.57 ± 1.34 | 0.47 ± 1.22 | 0.736 | 0.88 ± 1.45 | 0.76 ± 1.51 | 0.567 |
| **Weight-for-length Z-score*** | -1.28 ± 1.41 | -1.38 ± 1.44 | 0.767 | -1.17 ± 1.48 | -1.12 ± 1.57 | 0.818 |
| **Head circumference-for-age Z-score*** | 0.46 ± 1.36 | 0.45 ± 1.06 | 0.971 | 0.59 ± 1.21 | 0.82 ± 1.27 | 0.194 |
| **BW for gestational age <10^th^ centile (SGA) for RI groups, n (%)** | 12 (20.0%) | 6 (23.1%) | 0.695 | 112 (10.8%) | 8 (14.5%) | 0.371 |

* Mean ± SD; ** Median (range); ^†^ Mann-Whitney U test

**Abbreviations:** RI= Resistance index (of umbilical artery); y= years; n= number; M= male; F= female; w= weeks; g= grams; cm= centimetres; BW= birth weight; SGA= small-for-gestational age; AGA= appropriate-for-gestational age; SD= standard deviation
