# Supplementary material for: The ability of continuous-wave Doppler ultrasound to detect fetal growth restriction
Source: PLoS One. 2021 Aug 9;16(8):e0255960. doi: 10.1371/journal.pone.0255960 (PMC8351973; doi:10.1371/journal.pone.0255960)
Supplement: S3 Table — (DOCX) [file pone.0255960.s003.docx]

**Supplementary Table 3: Pregnancy and birth characteristics of the infant follow-up study compared to the Umbiflow^TM^ International** **participants, grouped by birth weight-for-gestational age categories.**

|  | **SGA** | |  | **AGA** | |  |
| --- | --- | --- | --- | --- | --- | --- |
|  | **Umbiflow International** | **UmbiBaby** | **P-value** | **Umbiflow International** | **UmbiBaby** | **P-value** |
|  | **(n=124)** | **(n=14)** |  | **(n=971)** | **(n=67)** |  |
| **Maternal age^*^, y** | 27.0 ± 5.5 | 27.1 ± 5.8 | 0.952 | 27.7 ± 5.1 | 29.3 ± 5.7 | **0.028** |
| **Gravidity**** | 2 (1-5) | 2 (1-5) | 0.953^†^ | 2 (1-12) | 2 (1-5) | 0.576^†^ |
| **Maternal HIV status positive, n (%)** | 30 (28.0%) | 6 (42.9%) | 0.216 | 296 (33.9%) | 19 (28.4%) | 0.338 |
| **Infant sex, M/F** | 66/58 | 8/6 | 0.768 | 486/485 | 33/34 | 0.896 |
| **Gestational age at birth*, w** | 39.6 ± 1.5 | 38.9 ± 1.2 | 0.059 | 39.2 ± 1.3 | 38.9 ± 1.2 | 0.053 |
| **Birth weight* (BW), g** | 2563 ± 260 | 2511 ± 250 | 0.473 | 3234 ± 383 | 3172 ± 441 | 0.266 |
| **Length*, cm** | 48.4 ± 2.3 | 48.2 ± 1.9 | 0.720 | 50.8 ± 2.6 | 50.4 ± 2.5 | 0.210 |
| **Head circumference*, cm** | 33.3 ± 1.5 | 33.7 ± 1.4 | 0.329 | 34.6 ± 1.5 | 34.6 ± 1.6 | >0.999 |
| **Weight-for-age Z-score*** | -1.77 ± 0.40 | -1.67 ± 0.22 | 0.360 | 0.07 ± 0.84 | 0.02 ± 0.87 | 0.650 |
| **Length-for-age Z-score*** | -0.49 ± 1.27 | -0.38 ± 1.21 | 0.753 | 1.04 ± 1.37 | 0.89 ± 1.36 | 0.386 |
| **Weight-for-length Z-score*** | -2.00 ± 1.28 | -1.83 ± 1.48 | 0.686 | -1.08 ± 1.47 | -1.08 ± 1.51 | >0.999 |
| **Head circumference-for-age Z-score*** | -0.50 ± 1.20 | 0.07 ± 1.13 | 0.094 | 0.73 ± 1.15 | 0.84 ± 1.19 | 0.466 |
| **Abnormal RI per SGA/AGA groups, n (%)** | 12 (9.7%) | 6 (42**.**9%) | **<0.001** | 48 (4.9%) | 20 (29**.**9%) | **<0.001** |

* Mean ± SD; ** Median (range); ^†^ Mann-Whitney U test

**Abbreviations:** y= years; n= number; M= male; F= female; w= weeks; g= grams; cm= centimetres; BW= birth weight; RI= Resistance index (of umbilical artery); SGA= small-for-gestational age; AGA= appropriate-for-gestational age; SD= standard deviation
